# Myelodysplastic Syndrome/Acute Myeloid Leukemia Arising in Idiopathic Erythrocytosis

**DOI:** 10.1155/2018/4378310

**Published:** 2018-02-22

**Authors:** Stephen E. Langabeer, Eibhlin Conneally, Catherine M. Flynn

**Affiliations:** ^1^Cancer Molecular Diagnostics, St. James's Hospital, Dublin 8, Ireland; ^2^Department of Haematology, St. James's Hospital, Dublin 8, Ireland

## Abstract

The term “idiopathic erythrocytosis (IE)” is applied to those cases where a causal clinical or pathological event cannot be elucidated and likely reflects a spectrum of underlying medical and molecular abnormalities. The clinical course of a patient with IE is described manifesting as a persistent erythrocytosis with a low serum erythropoietin level, mild eosinophilia, and with evidence of a thrombotic event. The patient subsequently developed a myelodysplasic syndrome (MDS) and acute myeloid leukemia (AML), an event not observed in erythrocytosis patients other than those with polycythemia vera (PV). Application of a next-generation sequencing (NGS) approach targeted for myeloid malignancies confirmed wild-type *JAK2* exons 12–15 and identified a common *SH2B3* W262R single-nucleotide polymorphism associated with the development of hematological features of myeloproliferative neoplasms (MPNs). Further NGS analysis detected a *CBL* L380P mutated clone expanding in parallel with the development of MDS and subsequent AML. Despite the absence of *JAK2*, *MPL* exon 10, or *CALR* exon 9 mutations, a similarity with the disease course of PV/MPN was evident. A clonal link between the erythrocytosis and AML could be neither confirmed nor excluded. Future molecular identification of the mechanisms underlying IE is likely to provide a more refined therapeutic approach.

## 1. Introduction

The causes of erythrocytosis are many and can be broadly divided into either primary or secondary forms. Primary causes are due to a defect intrinsic to the erythroid compartment of the bone marrow which leads to increased red cell generation, whereas secondary causes are due to factors external to the bone marrow that are produced in excess and drive red cell production [1]. The most common cause of acquired primary erythrocytosis is the myeloproliferative neoplasm (MPN) of polycythemia vera (PV) that is molecularly characterized by the *JAK2* p.V617F and exon 12 mutations [2]. Mutations of other genes in the erythropoiesis, oxygen sensing, and oxygen transport pathways are known to result in erythrocytosis; however, the underlying causes are unknown in a large number of cases, particularly those recognized as congenital erythrocytosis, and remain classified as idiopathic erythrocytosis (IE) [3]. Several clinical and biological differences exist between IE and PV, including a considerably lower risk of thrombotic events in IE patients compared to PV patients [4] with transformation to acute myeloid leukemia (AML) exceedingly rare in individuals with IE or other molecularly annotated forms of erythrocytosis [5]. A case is described in which application of the myeloid malignancy-targeted, next-generation sequencing (NGS) approach retrospectively provided insights into the molecular appearance of myelodysplastic syndrome (MDS)/AML in a patient with IE.

## 2. Case Report

**Figure 1:**
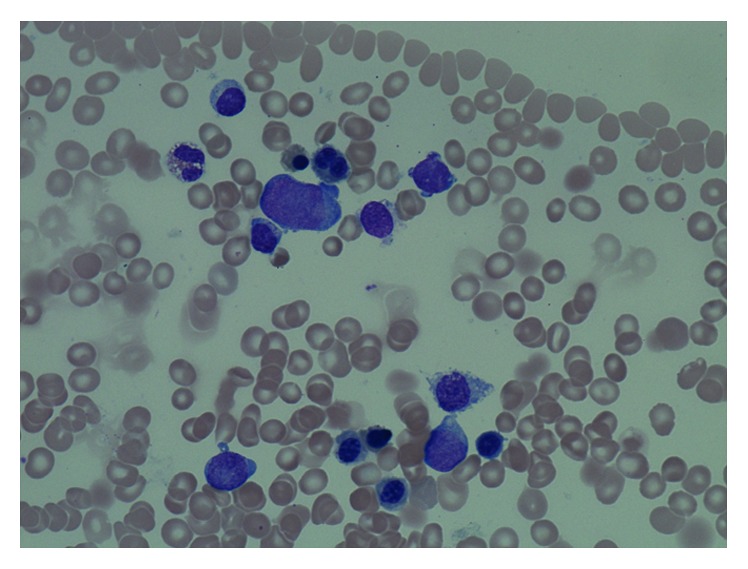
Bone marrow morphology at development of myelodysplasia/acute myeloid leukemia showing myeloblasts and dysplastic red blood cell precursors.

An overweight 62-year-old male with hypertension and hyperlipidemia presented with a hemoglobin level of 20.4 g/dL, hematocrit of 0.59, normal white cell and platelet counts, and a mild eosinophilia (Table 1). The patient had no clinical signs of PV, normal spleen size, normal oxygen saturation, no endogenous erythrocyte colony formation, no evidence of abnormal hemoglobin, and no family history of a hematological abnormality. The patient was managed with intermittent venesection alone for nine years with no evidence of the *JAK2* p.V617F mutations by allele-specific PCR or the *JAK2* exon 12 mutations by high-resolution melt curve analysis, excluding a diagnosis of PV [2]. At 149 months after diagnosis, thrombocytopenia (75 × 10^9^/L) and increasing eosinophilia (1.9 × 10^9^/L) were noted with a low serum erythropoietin (EPO) level of 0.6 IU/L (normal range 2.6–18.5 IU/L). At 154 months after diagnosis, the patient had temporoparietal stroke. Worsening pancytopenia at 157 months prompted bone marrow biopsy which demonstrated 4% myeloblasts (Table 1). Subsequent bone marrow investigations showed progressively increasing myeloblast cell numbers by both morphological and immunophenotypic evaluation with reduced dysplastic megakaryocytes, dyserythropoiesis with basophilic stippling, and binuclear red cell forms, all consistent with MDS progressing to AML (Figure 1). The karyotype at 159 months was normal. The patient was not fit for intensive treatment and was given best supportive care with EPO, intermittent red cell transfusions, and low-dose steroids but died of infection at 161 months.

A targeted NGS approach panel was retrospectively employed to detect mutations possibly contributing to the erythrocytosis and subsequent development of MDS/AML in archival peripheral blood or bone marrow DNA samples from 41, 149, and 161 months. Amplicon libraries covering thirty commonly mutated genes implicated in myeloid malignancies, either covering the entire coding region (*CALR*, *CEBPA*, *ETV6*, *EZH2*, *RUNX1*, *SH2B3*, *TET2*, *TP53,* and *ZRSR2*) or mutational hotspots (*ABL1*, *ASXL1*, *BRAF*, *CBL*, *CSF3R*, *DNMT3A*, *FLT3*, *GATA2*, *IDH1*, *IDH2*, *JAK2*, *KIT*, *KRAS*, *MPL*, *NPM1*, *NRAS*, *PTPN11*, *SETBP1*, *SF3B1*, *SRSF2,* and *U2AF1*), were generated. Sequencing was performed with Ion AmpliSeq™ methodology (Thermo Fisher Scientific, Paisley, UK). Calling of somatic mutations was achieved using an algorithm that excluded synonymous mutations, variants located within intronic or untranslated regions, and those present at a variant allele frequency (VAF) of <5%. A minimum target depth of coverage for variant calls was set at 500x as previously described [6, 7]. The nonsynonymous *SH2B3* exon 2, single-nucleotide polymorphism (SNP) W262R (c.784T>C, NP_005466.1, rs3184504) was present at all three time points analyzed at VAFs of approximately 50% (Table 1). No mutations were detected in *MPL* exon 10 or *CALR* exon 9. A single *CBL* L380P mutation (c.1139T>C, NP_005179.2) was detected in the bone marrow sample at 161 months. Reinterrogation of sequencing data revealed this *CBL* mutation to be present a year previously at a VAF of 3.6% (Table 1).

## 3. Discussion

Despite the absence of either the *JAK2* V617F or exon 12 mutations in the patient, a high degree of suspicion remained throughout the clinical course for a diagnosis of PV or “PV-like” MPN given the persistently raised hematocrit, the low serum EPO, a mild eosinophilia, and clinically a thrombotic episode (stroke). While NGS confirmed the absence of the *JAK2* V617F and exon 12 mutations, several alternative mutations of *JAK2* have been identified in sporadic cases of “PV-like” MPN and hereditary erythrocytosis [8–13], yet none were identified by NGS in exons 12–15 in the patient.

Of interest is the presence of the *SH2B3* W262R SNP. *SH2B3* (formerly *LNK*) encodes the *LNK* inhibitory adaptor protein that modulates thrombopoietin and erythropoietin signalling by interacting with *JAK2* and inhibiting downstream STAT activation. Disruption of this function by a mutant protein results in aberrant JAK-STAT signalling and cytokine responsiveness. Low frequency but recurrent, acquired, and germ line mutations of *SH2B3* have been reported in both sporadic and familial MPN, respectively, particularly in those cases and kindred identified with IE [14, 15]. While no somatic mutations were identified within the entire coding region of *SH2B3*, this patient was heterozygous (T/C) for the common W262R SNP [16]. This SNP and others within *SH2B3* have been shown to be associated with an increase in platelets, eosinophils, and elevated hemoglobin and hematocrit levels [17–22]. Furthermore, some recent evidence exists for the T allele of this SNP to be associated with the development of MPN and *JAK2* V617F-positive hematopoiesis [16, 21, 22]. Despite *in vitro* functional analysis of another nonsynonymous *SH2B3* SNP (E400K) in a patient with IE indicating no impairment of *SH2B3* inhibiting *JAK2*-STAT5 activation, subtle loss of function induced by *SH2B3* SNPs cannot be excluded [23]. While tempting to speculate a causal role for this *SH2B3* SNP in the development of erythrocytosis in this patient, no specific evidence exists for such an association, suggesting involvement of additional genetic and/or epigenetic events.

Transformation to AML is a recurrent event in PV with reported risks of 2.3–14.4% at ten years [24]. However, transformation to or development of MDS/AML in other forms of molecularly annotated erythrocytosis or IE is exceedingly rare [25]. A true transformation of the erythrocytosis could neither be confirmed nor excluded in this case due to the absence of a pre-MDS/AML marker of clonality with the possibility that the erythrocytosis and MDS/AML represent two unrelated pathologies. Activating mutations of *CBL*, a negative regulator of receptor tyrosine kinases, including the p.L380P detected in this case, is recurrent in myeloid malignancies and is associated with progression of MDS to AML [26].

In conclusion, we describe a patient with IE possessing a clinical similarity to PV in which there are persistent erythrocytosis, a thrombotic event, and the acquisition of somatic mutations that resulted in MDS/AML. Employment of an NGS gene panel specifically targeted for investigation of IE has recently demonstrated the benefits of this type of approach [27]. The potential exists for identifying those patients at increased risk of developing a myeloid malignancy, enabling refined counseling and therapeutic decision-making throughout the disease course.

## Figures and Tables

**Table 1:** Hematological indices and molecular analysis throughout the patient's clinical course.

Time (months)	Hb	HCT	RCC	PLT	WCC	Eos	BM blasts (IP/morphology)	Mutations detected (variant allele frequency)	
Dx	20.4	0.59	6.20	140	5.8	1.0	—	—	
41	16.2	0.49	6.29	158	6.8	0.9	—	*SH2B3* p.W262R (52.4%)	
149	19.7	0.58	6.78	75	8.1	1.9	—	*SH2B3* p.W262R (45.9%)	*CBL* p.L380P (3.6%)
157	8.9	0.26	2.62	112	4.0	0.2	4%/0%	—	
159	10.1	0.30	3.00	106	3.3	0.1	8%/6%	—	
161	10.1	0.29	3.19	35	6.1	0.4	15%/22%	*SH2B3* p.W262R (56.8%)	*CBL* p.L380P (37.2%)

Hb: hemoglobin (g/dL); HCT: hematocrit; RCC: red cell count (×10^12^/L); PLT: platelet count (×10^9^/L); WCC: white cell count (×10^9^/L); Eos: eosinophil count (×10^9^/L); BM blasts: bone marrow myeloblasts; IP: immunophenotyping; Dx: diagnosis.
